# Transition overtime in household latrine use in rural Bangladesh: a longitudinal cohort study

**DOI:** 10.1186/1471-2458-14-721

**Published:** 2014-07-15

**Authors:** Tahera Akter, Abu RMM Ali, Nepal C Dey

**Affiliations:** 1 Senior Research Associate, Environment Research Unit, Research and Evaluation Division, BRAC Centre, 75, Mohakhali, Dhaka 1212, Bangladesh; 2 Project Associate, Innovations for Poverty Action, Apt # 6B, House # 35, Road # 7, Block-G, Banani, Dhaka 1213, Bangladesh; 3 Research Fellow & Coordinator, Environment Research Unit, Research and Evaluation Division, BRAC Centre, 75, Mohakhali, Dhaka 1212, Bangladesh

**Keywords:** Transition, Sanitary latrine, Unsanitary latrine, Log-binomial regression, BRAC WASH

## Abstract

**Background:**

In a low-income country like Bangladesh, where the poverty rate is higher in rural compared to urban areas, the consistent use of sanitary latrines over time is a challenge. To address this issue, the Water, Sanitation, and Hygiene (WASH) program of the Bangladesh Rural Advancement Committee (BRAC) was devised to improve health of the rural poor through enhanced sanitation services, such as by providing loans or education. Sanitary latrine use in households and changes over time were assessed in this study.

**Methods:**

This was a longitudinal cohort study of the baseline, midline, and end line status of the WASH project. Households assessed in all three rounds of surveys (26,404 in each survey) were included in the analysis. Thirty thousand households from 50 *upazilas* (sub-districts) were selected in two stages: i) thirty villages were selected from each of the 50 *upazilas* by cluster sampling, and ii) twenty households were chosen systematically from each selected village. A female member capable of providing household-level information was interviewed from each house using the pre-tested questionnaire. Spot observations of some components were made to assess the quality of sanitary latrine use. The adjusted log-binomial regression was performed and risk ratios with 95% confidence intervals were estimated for sanitary latrine use. Data were analyzed using Statistical Package for the Social Sciences (SPSS) and Stata software.

**Results:**

The use of sanitary latrines by households increased significantly from the baseline (31.7%) to midline (41.5%) and end line (57.4%) assessment points. The proportion of physically verified clean latrines increased significantly from 33.4% at baseline to 50.8% at the midline and 53.3% at the end line. Analysis of changes in latrine-use showed that 73.3% of the baseline latrine-using households continued to do so at the end line, while the rest switched to unsanitary practices. Households with better socioeconomic status were more likely to use sanitary latrines.

**Conclusion:**

There are improvements in ownership and use of sanitary latrines by households over the years in WASH intervention areas. However, switching of some households from sanitary to unsanitary latrines remains a matter of concern regarding sustainability.

## Background

Globally, the quality of drinking water seems to be better than that of sanitation. It is often reported that the proportion of households with access to safe drinking water is on track to meet the Millennium Development Goals (MDGs), while the proportion of those with access to proper sanitation is often said to be “lagging behind water supply” [1]. Currently, of the 2.6 billion people that lack access to hygienic sanitation worldwide, two-thirds live in Asia and sub-Saharan Africa [2]. In developed countries, 99% of the population has access to hygienic sanitation, while in developing countries the proportion is only 53%. Within developing countries, there is a wide gap between urban (71%) and rural (39%) sanitation coverage. Currently, the majority of the people who lack sanitation live in rural areas, and globally, eight out of ten users of unhygienic sanitation facilities, and six out of seven people who defecate in the open, live in rural areas [1].

In Bangladesh, almost one-third of the population lives below the poverty line. The poverty rate in rural areas (36%) is higher than urban regions (28%) [3]. These conditions result in more people suffering from diseases caused by a lack of clean drinking water and sanitation [4]. Among the poorest, almost one in three people defecates in the open, making the environment unsafe [5]. Thus, unhygienic sanitation practices threaten public health, spread diarrhea, typhoid, and other diseases through pathogens in feces. The World Health Organization (WHO) estimates that 1.5 million children die from diarrheal diseases each year worldwide, with 88% of these deaths occurring due to inadequate sanitation, improper hygiene, and unsafe drinking water [6]. In Bangladesh, the leading cause of death among children below the age of five (including 20% of all infant deaths) is diarrheal disease [7].

The government of Bangladesh had set a national target of 100% sanitation coverage by 2013 as a step towards achieving the MDGs. A national sanitation campaign involving people from all strata of society was launched in 2003, with the aim of improving sanitation coverage in the country [8]. In order to achieve this goal, non-governmental organizations (NGOs) and the private sector joined the government to implement the water and sanitation program [9]. BRAC initiated a comprehensive WASH intervention program in 2006. The initiative covered 150 *upazilas* throughout the country, and was aimed at improving the health of the rural poor through provision of safe drinking water, access to sanitation services, and promotion of safe hygiene behavior in all economic groups [10]. The households were classified as ultra-poor, poor, and non-poor as per the following criteria of the BRAC WASH program: households that owned less than 404.7 m^2^ of land, had no fixed source of income, or were headed by a female were classified as “ultra-poor”; households with land holdings between 404.7 m^2^ and 4047 m^2^ and/or sold manual labor for a living were classified as “poor”; and households that did not fall into either of the above categories were classified as “non-poor”.

BRAC WASH latrine support is provided according to the households’ economic status. If the sanitation coverage of a village is greater than or equal to 80%, the WASH program provides free latrine support to the ultra-poor in the village to enable the village attain 100% sanitation coverage. If sanitation coverage of a particular village is less than 80%, then the ultra-poor and poor are given a subsidy to procure sanitary latrines. All economic groups (ultra-poor, poor, and non-poor) are given hygiene education through motivational cluster meetings.

Village WASH committees (VWCs) are formed through a community participatory process to facilitate intervention activities and improve the overall WASH situation in the selected villages. A VWC consists of 11 members (six women and five men) representing different segments of the community, such as local elite/leaders, school/madrasa teachers, adolescent girls, women, and micro-credit borrowers. The committee meets once a month for the first six months to scrutinize the activities of that month and to prepare a work plan for the next month. After this period, the committee meets every two to three months to assess the water and sanitation situation of the village and identify issues requiring urgent action. On average, there is one VWC for every 200 households. Some of the major tasks of the VWC are to make arrangements to install new sanitary latrines, convert unhygienic latrines to sanitary ones by changing water seals and installing tubewells, and arranging educational activities; these include health forums, folk songs, street plays, and film and video shows to increase awareness about hygienic behavior. The VWC identifies community water sources, collects money, and monitors usage and maintenance of sanitary latrines. Members of the BRAC staff liaise with government and union council representatives to make water and sanitation-related hardware available to the community. Voluntary health workers, program organizers (POs), program assistants (PAs), and managers are assigned by BRAC to implement intervention activities. Senior-level staffs from regional and head offices are appointed to supervise the implementation of the intervention protocols.

The Research and Evaluation Division (RED) of BRAC conducted a baseline survey from November 2006 to June 2007 to understand the pre-program sanitation status. A midline survey was carried out two years after the baseline survey (from April-June 2009) to assess the change in status of various intervention components. The end line evaluation of the impact of the BRAC WASH program on various aspects of water, sanitation, and hygiene was conducted between December 2010 and March 2011.

A number of studies have been conducted on sanitation-related issues, such as assessment of health and sanitation status by household characteristics [4,11,12], improvement in quantity and quality of sanitation coverage [13], and the role of NGOs in improving sanitation status [14]. Yusuf and Hussain have assessed the socioeconomic status of households’ having their own sanitary latrines [15]. The present study attempts to explore changes over time in households’ latrine use from sanitary to unsanitary practices, or vice-versa. We have also explored the probability and quality of sanitary latrine use throughout this study.

## Methods

### Study design

This was a longitudinal cohort study, covering the baseline (2006), midline (2009), and end line (2011) status of the project. The time interval between each of the three surveys was determined prior to the commencement of the five-year intervention program. A preparatory phase of six months was implemented before the project started, followed by three intervention periods of 1.5 years each. The intervention was carried out in 150 *upazilas* in three phases, each of six-month duration. Each phase covered 50 *upazilas. Upazilas* identified as low-performing areas in terms of water, sanitation, and hygiene coverage compared to national averages were selected for intervention. The baseline, midline and end line surveys were conducted in 50 *upazilas* of the first phase where the BRAC WASH program has been offering interventions since 2006.

### Sample size and sampling

Six hundred households from each of the 50 *upazilas* were sampled for each survey, giving a total sample size of 30,000 households. The sample size was determined using the multi-stage sampling technique, where each *upazila* was considered as a cluster. Considering a significance level at 5%, with admissible error of 5% and design effect of 1.5, the estimated sample size for the survey was 576 households for each *upazila*, which was rounded to 600 for distributive convenience. These 600 households were distributed among thirty villages of each *upazila*. The interval sampling method was used to select the villages. In the first stage, all villages within an *upazila* were listed. The interval size was then calculated by dividing the total number of villages within an *upazila* by thirty. The first village was selected randomly from the first interval to avoid systematic bias, and the consecutive villages were selected as per the estimated interval size. The interval sampling technique was used again to select twenty households from each village.

### Operational definition of a sanitary latrine

According to the Local Government Division (LGD), a sanitary/hygienic latrine should have the following characteristics: (i) feces should be sequestered from the environment; (ii) the passage between the squat hole and the pit must be sealed to effectively block flies and other insect vectors, thereby preventing disease transmission; and (iii) the latrine should be odor-free and clean, such that its continued use is encouraged [16]. The BRAC WASH program followed the government definition of hygienic latrines, but did not put a limit on the number of households sharing a latrine.

### Data management and analysis

Trained field interviewers collected data from households through face-to-face interviews using a pre-tested structured questionnaire. In all, 96 interviewers were employed, trained, and divided into 12 groups for data collection in the field. Each group had one supervisor. Self-reported sanitation-related data, including type of latrine used, ownership of the latrine, source of finance used for latrine installation, presence of water seal, and frequency of latrine cleaning, were collected. Information regarding socioeconomic status, such as number of members in the household and age, gender, main occupation, education, of each member, was also recorded. The quality of sanitary latrines was checked by spot observation of several parameters, including level of cleanliness of latrine, presence or absence of unpleasant odor, visibility of fecal matter, and availability of water and slippers in or near the latrine.

Field interviewers were given adequate training on data collection before commencement of the fieldwork. A training manual with instructions about data collection procedures was developed and used as a reference in the field. The interviewers worked in teams of about eight. A female member capable of providing household-level information was interviewed from each house using the pre-tested questionnaire. To ensure completeness and consistency, the interviewers were instructed to complete and crosscheck each other’s questionnaire. The field supervisor in each team re-checked 5% of previous week’s completed questionnaires. Field managers also checked the quality of the interviews by randomly checking 12 completed questionnaires on a particular day and also visiting the respective households to verify the answers to some of the questions. Whenever any inconsistency was identified, a re-interview was conducted to make the necessary corrections. The authors also visited the field regularly to check whether the data collection was being carried out as instructed.

The completed questionnaires were edited for completeness and consistency at the BRAC Head Office by a group of trained field interviewers. The data were entered into a computer and cleaned using the SPSS software under the supervision of the authors. The analysis was performed on matched households that were included in all the three surveys (26,404 households in each survey).

### Statistical analysis

In an analysis of the common outcome, as use of sanitary latrines in our study, the log-binomial regression was recommended for the estimation of risk ratios (RRs) and 95% confidence intervals (CIs). The interpretation of odds ratio (OR) as RR was not considered appropriate because OR overstated RR [17]. A database of dichotomous variables was created to simulate a cohort study in which a common outcome ‘sanitary latrine use’ (over 50% prevalence) was related to several independent predictors, such as exposure to schools, NGOs, and media (radio, TV, etc.) at home. In addition to these exposure factors, the households’ economic status and survey years were also included in the model. A score of ‘1’ was assigned to the groups exposed to the predictor factors, while the non-exposed were denoted as ‘0’. Statistical analysis was performed using Stata software. The results were expressed as RRs with 95% confidence intervals (CIs). The analysis included robust cluster variance at the village level to estimate unbiased standard errors [18].

### Ethical issues

BRAC RED gave ethical approval to the study. Permission to conduct this research was obtained from the BRAC WASH program. Informed verbal consent was taken from each respondent after reading out the consent form and explaining the general purpose of the study. Each respondent was assured that she could withdraw from the interview at any point, and that refusal to participate in the study would not affect her receiving any services from BRAC. Strict confidentiality was maintained in data handling. The name and identity of the respondent were not disclosed while reporting personal information.

## Results

### Background characteristics of the study samples

A total of 29,985 households at baseline, 29,885 households at the midpoint, and 26,404 households at the end point were included in the survey. Some households could not be included at the follow-up surveys due to unavailability, migration, or death of the members. Sampled households in the study area had an almost equal proportion of male and female members (50.4% vs. 49.6%). In the end line survey, background data showed that the majority of heads of households were non-poor (59%) and 55.3% had attended school at some point (Table 1). About one-third of the respondents were involved in agricultural work, 31% worked as day laborers, and 13% were involved with businesses. Over half of the households (56.1%) were not members of any NGO (nor had access to services provided by NGOs). About 37.5% of the respondents had access to media at home by radio and/or TV, while the majority did not own either (62.5%).

**Table 1:** Socioeconomic profile of sample households in the survey years (%; N = sample size)

**Indicators**	**BL %(N)**	**ML %(N)**	**EL %(N)**
**Education of head of the household**			
Ever schooled	55.4(14629)	54.6(14410)	55.3(14591)
Never schooled	44.6(11994)	45.4(11994)	44.7(11813)
**NGO membership of household**			
Yes	45.6(12016)	47.5(12489)	43.9(11477)
No	54.4(14327)	52.5(13800)	56.1(14684)
**Main occupation of head of the household**			
Agriculture	33.2(8778)	32.7(8622)	33.4(8827)
Labor	32.6(8598)	30.5(8047)	30.9(8150)
Service	6.5(1707)	5.9(1571)	6.1(1623)
Business	16.9(4474)	15.8(4168)	14.5(3821)
Household work	7(1846)	10.4(2735)	9.5(2505)
Disabled	2.2(578)	3(781)	3.8(1015)
Others	1.6(423)	1.8(480)	1.7(462)
**Economic status of head of the household**			
Ultra-poor	18.8(4959)	18.8(4959)	16.8(3361)
Poor	26.9(7115)	26.9(7115)	24.2(4964)
Non-poor	54.3(14330)	54.3(14330)	59(18079)
**Access to media at home (radio, TV)**			
Yes	37.4(9884)	37.9(9994)	37.5(9903)
No	62.6(16520)	62.1(16410)	62.5(16501)

BL = Baseline, ML = Midline, EL = End line.

### Types of latrines used in households

**Figure 1:**
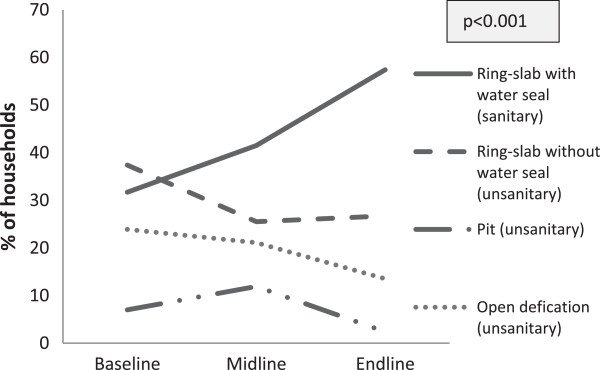
Households classified by the type of latrine used (%).

**Figure 2:**
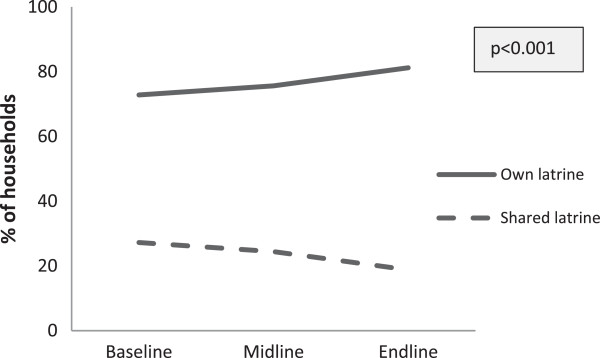
Households classified as per ownership of sanitary latrines (%).

The use of sanitary latrines by households increased significantly over the years, growing from 31.7% at baseline to 41.5% at the midline and 57.4% at the end line (p < 0.001; Figure 1). The proportion of households using ring-slab latrines without a water seal decreased from 37.4% at baseline to 25.5% at the midline and 26.7% at the end line. The proportion of pit latrine (open pits or a hole in the ground) users was higher at the midline survey (11.9%) but declined at the end line (2.4%), compared to baseline (7%). The proportion of people defecating in the open significantly reduced from baseline (23.9%) to the midline (21.1%) and end line (13.5%).The status of the households’ ownership of sanitary latrines showed significant changes across the surveys in intervention areas. The proportion of households that owned latrines increased significantly from baseline (72.8%) to the midline (75.6%) and end line (81.2%), while the proportion of shared latrines decreased significantly from 27.2% at baseline to 24.4% at the midline and 18.8% at the end line (Figure 2).

### The quality of sanitary latrines used

The quality of sanitary latrines was measured in terms of some physically verifiable indicators of cleanliness. As per the BRAC WASH program, a clean latrine was defined as one that did not produce a foul smell and had no flies or visible traces of fecal matter in/around it [19]. Other indicators used to measure the quality of sanitary latrines were the presence of a fence around the latrine and availability of water and slippers in or near the latrine. These indicators imply hygienic use of latrines. The quality of sanitary latrine use improved significantly over the intervention period in terms of latrine cleanliness (baseline 33.4%, midline 50.8%, end line 53.3% [p < 0.001]); availability of water (baseline 32.7%, midline 37.8%, end line 38.7%); and availability of slippers (baseline 4.4%, midline 8.2%, end line 13.5%) (Table 2). The proportion of latrines with unpleasant odor reduced to 48% at the midline and 50.1% at the end line compared with a baseline value of 62.9%. The indicator of visible fecal matter left in the latrine also showed a trend of decline similar to that of unpleasant odor.

**Table 2:** Indicators for quality of sanitary latrines (%; N = sample size)

**Is the latrine clean?**						
				**p value**		
	**BL**	**ML**	**EL**	**BL vs ML**	**ML vs EL**	**BL vs EL**
YES (%)	33.4	50.8	53.3	0.000	0.000	0.000
N	16822	18101	21519			
**Is there any unpleasant odor coming from the latrine?**						
YES (%)	62.9	48	50.1	0.000	0.000	0.000
N	16822	18101	21519			
**Is there any fecal matter left in the latrine?**						
YES (%)	48.2	35.2	37.9	0.000	0.000	0.000
N	16822	18101	21519			
**Is there a fence around the latrine?**						
YES (%)	98.9	98.1	99.1	0.000	0.000	0.065
N	16822	18101	21519			
**Is water available in and/or near the latrine?**						
YES (%)	32.7	37.8	38.7	0.000	0.054	0.000
N	16822	18101	21399			
**Is a pair of slippers present in or near the latrine?**						
YES (%)	4.4	8.2	13.5	0.000	0.000	0.000
N	16822	18101	21519			

BL = Baseline, ML = Midline, EL = End line.

### Transitions in sanitary latrine use

The transition matrix (Table 3a) shows the proportion of households that switched their sanitation practices during the period between baseline and midline surveys. Of 31.7% households using sanitary latrines at baseline, 66.5% continued the practice up to the midline, but the rest switched to other unsanitary practices, such as ring-slab latrines without water seals (18.1%) and open defecation (7.1%). Among the 37.4% households that used ring-slab latrines without water seals at baseline, 35% had shifted to sanitary latrine practices by the midline survey, while the rest continued other unsanitary practices, such as use of ring-slabs without water seals (36.6%), pits (14.7%), and open defecation (13.6%). Of pit latrine users at baseline, 25.4% had shifted to sanitary practices, while 26.7% had adopted ring-slab latrine without water seals, and 20.2% had switched to open defecation by the midline survey. Out of 23.9% households who used to defecate in the open at baseline, 23.7% had shifted to sanitary practices by the midline, while just over half (51.2%) continued with the same defecation practice.

**Table 3:** Transition matrix of latrine use in households (%; N = Sample size)

**(a) from BL to ML**						
**BL**		**ML**				
**Types of latrines %(N)**		**Sanitary**	**Ring-slab (no WS)**	**Pit (open)**	**Open defecation**	**Total**
**Sanitary**	31.7(8370)	66.5(5566)	18.1(1515)	8.3(695)	7.1(594)	100(8370)
**Ring-slab (no WS)**	37.4(9875)	35.1(3466)	36.6(3614)	14.7(1452)	13.6(1343)	100(9875)
**Pit (open)**	7(1848)	25.4(469)	26.7(493)	27.7(512)	20.2(373)	100(1848)
**Open defecation**	23.9(6311)	23.7(1496)	17.4(1098)	7.7(486)	51.2(3231)	100(6311)
**Total**	100(26404)					
**(b) from BL to EL**						
**BL**		**EL**				
**Types of latrines %(N)**		**Sanitary**	**Ring-slab (no WS)**	**Pit (open)**	**Open defecation**	**Total**
**Sanitary**	31.7(8370)	73.3(6135)	19.6(1641)	1.4(117)	5.7(477)	100(8370)
**Ring-slab (no WS)**	37.4(9875)	52.4(5175)	37.2(3674)	3(296)	7.4(731)	100(9875)
**Pit (open)**	7(1848)	46.6(861)	33.5(619)	11.1(205)	8.8(163)	100(1848)
**Open defecation**	23.9(6311)	46.4(2928)	17.8(1123)	2(126)	33.8(2133)	100(6311)
**Total**	100(26404)					

BL = Baseline, EL = End line, WS = Water seal.

The transition matrix (Table 3b) shows the proportion of households who changed their sanitation practices during the period between the baseline and end line surveys. Out of 31.7% households using sanitary latrines at baseline, 73.3% continued with this practice, while the rest switched to other unsanitary practices (ring-slab without water seals, 19.6%; pit latrines, 1.4%; and open defecation, 5.7%) by the end line survey. Of the 37.4% households using ring-slab latrines without water seals at the baseline survey, more than half (52.4%) had shifted to sanitary latrine practices by the end line, while the rest continued with other unsanitary practices (ring-slab without water seals, 37.2%; pit latrines, 3%; and open defecation, 7.4%). Out of the 7% of households that used pit latrines at baseline, 46.6% shifted to sanitary latrine practices, while the rest continued with unsanitary practices (ring-slab latrines without water seals, 33.5%; pit latrines, 11.1%; and open defecation, 8.8%). Among the 23.9% households who reported defecating in the open at baseline, 46.4% shifted to sanitary latrine practices, while the rest continued with unsanitary practices (ring-slab without water seals, 17.8%; pit latrines, 2%; and open defecation, 33.8%) up to the end line survey.

### Relative probability of sanitary latrine use

The results demonstrate that use of sanitary latrines increased 1.30 times (95% CI: 1.26-1.34) by the midline (2009) and 1.67 times (95% CI: 1.62-1.72) by the end line (2011) compared to baseline use (2006) (Table 4). Those who had exposure to education were 1.27 times more likely to use sanitary latrines compared to those without schooling (95% CI: 1.24-1.29). Those who had access to radio and/or TV at home were 1.37 times more likely to use sanitary latrines compared to those with no access to media (95% CI: 1.34-1.40). Poor and non-poor households were 1.06 times (95% CI: 1.02-1.10) and 1.23 times (95% CI: 1.29-1.27) more likely to use sanitary latrines, respectively, compared to the ultra-poor. However, households affiliated with NGOs were negatively associated with the use of sanitary latrines (RR 0.93, 95% CI: 0.91-0.94).

**Table 4:** Association between sanitary latrine use and its predictors

**Indicator**	**Risk ratio**	**95% CI**	**Semi robust SE**	**p value**
**Survey year**				
Baseline	1			
Midline	1.30	1.26-1.34	.0142884	< 0.001
End line	1.67	1.62-1.72	.016937	< 0.001
**Household economic status**				
Ultra-poor	1			
Poor	1.06	1.02-1.10	.0161791	< 0.001
Non-poor	1.23	1.19-1.27	.0162596	< 0.001
**Education**				
Never schooled	1			
Ever Schooled	1.27	1.24-1.29	.0109777	< 0.001
**NGO membership**				
No membership	1			
Member of any NGO	0.93	0.91-0.94	.0070973	< 0.001
**Access to media at home (radio, TV)**				
No access to media	1			
Access to media	1.37	1.34-1.40	.0108278	<0.001

SE = Standard error.

## Discussion

The present study investigated the trends in latrine use in the rural poor by analyzing various parameters, such as types of latrines used at the household level, their ownership and hygienic use, and transition to, and likelihood of using, sanitary latrines. This study showed that 57.4% of the sampled households were using sanitary latrines in 2011. According to the Bangladesh Bureau of Statistics (BBS) and UNICEF, the proportion of households using sanitary latrines in rural areas was 49.9% in 2009 [20]. The LGD pointed out that one possible reason for variations in sanitation coverage could be a lack of understanding about the role of water seals in hygiene [21]. Consistent with our findings, improvement in the use of sanitary latrines over the years has been reported in a number of studies, despite the variations in sanitation coverage [22,23].

The proportion of households using ring-slab latrines without water seals decreased significantly from baseline to midline, but increased slightly from midline to end line. A tendency to break the water seal has been observed in a few households in the intervention areas, mainly due to water shortage or lack of awareness. This is a hindrance to improving sanitation coverage. If effectively motivated, households that have latrines with broken water seals can be converted into households with an intact water seals, thus increasing the overall sanitation coverage. According to some users, water seal latrines are not convenient to use and maintain [24]. Substantial amounts of water are required to clean the latrine after use and carrying water from its source is often difficult. Hence, people sometimes break the water seal to reduce the amount of water required for flushing. However, they are unaware that insects can enter the latrine pit through a broken water seal [5].

The quality of sanitary latrines improved from baseline to end line, but not all the sanitary latrines were being used hygienically. Improvement in hygienic use of latrines was found in the WASH intervention areas where households were strongly motivated by training and door-to-door visits by village WASH committees [25]. Increasing in the number of latrines alone cannot lead to improved public health if those latrines are not maintained and used hygienically [13]. Therefore, emphasis was placed on changing the households’ behavior, such that quality of sanitary latrines improved.

The proportion of households switching to sanitary practices was higher than that of those switching to unsanitary ones, implying that people were increasingly adopting hygienic practices. Shared latrine users who do not have their own latrines may at times defecate in the open, as many households whose latrines become unsuitable for use over time and are not repaired or replaced. Rural households also tend to slip back into old, unsanitary habits very quickly if new latrines become blocked, broken, or smell bad and if timely guidance and encouragement are not provided [13]. Users of shared latrines and households without latrines are most likely to practice open defecation.

Households with low socioeconomic status, such as ones in which the members are poor, never schooled, and never exposed to the media at home, are less likely to use sanitary latrines. A study by Yusuf and Hussain reported that people do not always use the sanitary latrines provided to them, since this habit is influenced by socio-cultural conditions and education [15]. Another study found that the poor often showed lack of interest in obtaining latrines on loan, as they were reluctant to spend money and desired to own latrines free of cost. They often expected BRAC to defer their rule of providing free latrines only to the ultra-poor [26]. Among the sampled population, affiliation of households with NGOs did not have any influence on the use of sanitary latrines. One possible reason could be the high proportion of non-poor households among those without affiliation to any NGO. The number of households switching to sanitary practices increased over the years despite the majority of the sampled population not having an association with any NGO. Although the present study did not find a positive correlation between affiliation with NGOs and improved sanitary practices, others have reported differently; Hadi and Nath found that assistance from NGOs, along with services such as loan support, group formation, and training, helped to change the sanitary practices of people by increasing awareness as well as financial capacity [27]. Moreover, a recent study conducted by Rabbi and Dey (2013) found a strong association between good hygiene practices and NGO membership [28]. The unmet need for safe sanitation for the poor can be met by NGO-led development program such as credit schemes, which could play a significant role in improving the sanitation situation in rural Bangladesh [29].

A major limitation of this study is the absence of a control group, which may raise the question of whether or not the observed changes in sanitary practices are due to WASH interventions. Our attribution of all changes in latrine coverage and use to the BRAC program makes the assumption that in the absence of the program there would be no change in latrine conditions in this population over the study period. Without a control group, we cannot definitively conclude whether the estimated effects are an over-estimate or an under-estimate of the program’s impact. However, we expect that the differences between survey rounds are a reasonable estimate of the program’s effect on sanitation conditions because data gathered from the same households in midline and end line surveys clearly demonstrate the positive changes in latrine coverage and use, while considering the baseline survey prior to the WASH intervention as a reference point for the analysis. Households in WASH intervention areas are more likely to own and use sanitary latrines compared to others [14]. Moreover, no other NGO has undertaken a similar, large intervention related to water, sanitation, and hygiene in BRAC WASH program areas during the same period.

## Conclusion

Our data demonstrate the improvement in ownership and use of sanitary latrines at the household level in WASH intervention areas. However, the incidence of some households shifting from sanitary to unsanitary practices over the years raises concerns regarding sustainability. Several factors such as poverty, lack of awareness, and water shortage induce households to adopt unsanitary practices. Manifestations of these factors are observed as breaking of water seals and occasional or regular defecation in the open. Despite these challenges, the condition of sanitation is improving. In order to further improve it, increased awareness of sanitary latrine use, periodic home visits to monitor usage, and maintenance of latrines are imperative.

## Competing interests

The authors declare that they have no competing interests.

## Authors’ contributions

TA contributed to the conception, study design, data collection, analysis, interpretation of data, and writing the manuscript as the lead writer. ARMMA contributed to the study design, data collection and analysis. NCD contributed to the study design, data collection and commented on the draft manuscript. Authors read and approved the final manuscript.

## Pre-publication history

The pre-publication history for this paper can be accessed here:

http://www.biomedcentral.com/1471-2458/14/721/prepub
